# Human Papillomavirus (HPV) Infection and Risk Behavior in Vaccinated and Non-Vaccinated Paraguayan Young Women

**DOI:** 10.3390/pathogens13030209

**Published:** 2024-02-27

**Authors:** María Liz Bobadilla, Verónica Villagra, Héctor Castro, Marta von Horoch, Soraya Araya, Gerardo Deluca, Vanessa Salete de Paula

**Affiliations:** 1 Laboratory of Immunology, Central Laboratory of Public Health, Minister of Public Health and Social Welfare, Asunción 1429, Paraguay; v.villagra2@gmail.com; 2 Laboratory of Molecular Virology and Parasitology, Oswaldo Cruz Institute, Oswaldo Cruz Foundation, Rio de Janeiro 21040-360, Brazil; vdepaula@ioc.fiocruz.br; 3 Expanded Immunization Program, Minister of Public Health and Social Welfare, Asunción 1429, Paraguay; hector.castro@mspbs.gov.py (H.C.); martavhv@gmail.com (M.v.H.); angelaarayayampey@gmail.com (S.A.); 4 Molecular Applications Laboratory, Faculty of Medicine, Northeast National University, Corrientes 1240, Argentina; delucagd@gmail.com

**Keywords:** cervical cancer, human papillomavirus, HPV vaccination, sexual behavior, vaccine impact, Paraguay

## Abstract

Cervical cancer is a global health concern and ranks fourth among the most prevalent cancers in women worldwide. Human papillomavirus (HPV) infection is a known precursor of cervical cancer and preventive measures include prophylactic vaccines. This study focused on sexually active Paraguayan women aged 18–25 years, exploring the intersection of HPV vaccination and sexual behavior. Among 254 participants, 40.9% received the Gardasil-4 vaccine, with no significant differences in sexual behavior between the vaccinated and unvaccinated sexually active groups. However, a notable decrease in the prevalence of HPV among the vaccinated women highlights the efficacy of this vaccine in reducing infections. The prevalence of any HPV type was 37.5% in vaccinated participants compared to 56.7% in unvaccinated participants (*p* = 0.0026). High-risk HPV types showed a significant difference, with a prevalence of 26.0% in vaccinated women compared with 52.7% in unvaccinated women (*p* < 0.001). Although a potential decline in genital warts was observed among the vaccinated individuals, statistical significance (*p* = 0.0564) was not reached. Despite the challenges in achieving high vaccination coverage, the observed reduction in HPV prevalence underscores the importance of ongoing monitoring, healthcare professional recommendations, and comprehensive risk management. These findings contribute to dispelling concerns about HPV vaccination influencing sexual behavior, advocating further large-scale research to explore the impact of vaccines on various HPV types and potential cross-protection.

## 1. Introduction

Cervical cancer is the fourth most prevalent cancer in women worldwide, with 604,127 newly reported cases and 341,831 deaths reported in 2020 [1]. Human papillomavirus (HPV) is a well-established cause of cervical cancer. HPV is the most prevalent sexually transmitted infection, and although most infections resolve spontaneously, persistent HPV infections can lead to precancerous and cancerous lesions.

Prevention of cervical cancer involves the early identification of precursor lesions and cancer as well as primary prevention through the administration of prophylactic vaccines. The World Health Organization (WHO) recommends including the HPV vaccine in the national immunization schedule, and HPV vaccination as a foundational pillar of the WHO global strategy to accelerate the elimination of cervical cancer as a public health problem [2]. 

Currently, there are six licensed prophylactic HPV vaccines: three bivalent, two quadrivalent, and one nonavalent. Numerous clinical trials have revealed that Cervarix (HPV types 16/18) and Gardasil-4 (HPV types 6/11/16/18) are safe and effective in preventing infections with these included types and associated cervical lesions and cancers [3,4,5,6,7]. Gardasil-4 also prevents anogenital warts [3,4,8,9,10] and Garadasil-9 extends its protective coverage to additional types, specifically 31, 33, 45, 52, and 58 [11]. Little information is available on the newer HPV vaccines [12]. 

By October 2023, 139 countries had introduced HPV vaccines into their national routine immunization schedules [13]. In Paraguay, the quadrivalent HPV vaccine was incorporated into the national immunization program in 2013, with a focus on girls aged 9–10 years. The immunization schedule involved doses administered at 0, 1, and 6 months, and vaccination was conducted through school-based programs. In 2017, following the updated HPV vaccine recommendations of the WHO Strategic Advisory Group of Experts, Paraguay transitioned to a two-dose regimen for girls aged 9–14 years. The vaccination schedule was set at 0 and 6 months in alignment with the new guidelines. This expanded immunization strategy involves both school- and facility-based vaccination approaches [14,15]. 

Because 90% of HPV vaccination coverage could contribute to the eradication of cervical cancer, low HPV vaccination rates remain a major challenge in several countries that have incorporated HPV vaccines into their regular immunization programs [16,17,18,19]. There have been questions regarding whether HPV vaccination encourages riskier sexual activity, which is one of the main barriers to the optimal acceptance of the vaccine [20,21,22].

Evidence from high-income countries indicates that concerns regarding an elevated risk of sexual behavior or promiscuity following HPV vaccination are unfounded [23,24,25,26,27,28,29]. Nevertheless, in Paraguay, where HPV vaccination programs have been in place since 2013, there is still a lack of evidence suggesting that HPV vaccination has led to increased sexual behavior among young women. The objective of this study was to investigate the sexual behavior and associated variables among sexually active young Paraguayan women, exploring their relationship with the uptake of HPV vaccination. 

## 2. Materials and Methods

### 2.1. Study Population 

The study population consisted of young women aged 18–25 years who visited the Central Laboratory of Public Health (LCSP) between May 2020 and September 2023. Participants were invited to local health facilities and higher education institutions through social networks and fliers. The inclusion criteria were women who were sexually active and not pregnant. The exclusion criteria were a history of hysterectomy or had never had sexual intercourse. All invited women who agreed to participate in this study signed a free informed consent form and completed a self-administered questionnaire that included basic demographic data, HPV vaccination status, and determining factors of HPV infection (age, origin, occupation, educational level, age at first sexual intercourse, number of sexual partners in the previous 12 months, methods of contraception, sexual practices, alcohol consumption, and tobacco use). The vaccination status was self-reported by vaccine recipients, as the electronic vaccination record was implemented in 2021 for COVID-19 vaccines and in 2022 for other vaccines. Before this, records were maintained through physical forms and transcribed onto vaccination cards, which were often lost, posing challenges for reliable verification. All vaccinated participants received immunization with the quadrivalent vaccine Gardasil-4 (HPV types 6/11/16/18; Merck, NJ, USA), as part of the national immunization program. The samples were processed at the LCSP, which is the National Reference HPV Laboratory in Paraguay. 

### 2.2. Clinical Specimen Collection

After verbal and written/illustrated instructions, cervical samples were self-collected using an Evalyn Brush (Rovers Medical Devices, Oss, The Netherlands). The samples were stored at 8 °C. The brush head was vigorously shaken inside vials containing 2.0 mL of sterile phosphate-buffered saline (PBS) to facilitate the separation of the collected cells. Subsequently, the brush heads were discarded, and the samples were stored at 8 °C until processing, at a time not exceeding 72 h. Each sample was assigned an anonymous and unique participant code to preserve the identity of each participant.

### 2.3. DNA Extraction from Cervical Samples

One milliliter aliquots were centrifuged at 12,000 rpm for 10 min to obtain a pellet of exfoliated cervical cells. After removing the supernatant, 1.0 mL of sterile water was added to the suspension, and the cells were washed. After another round of centrifugation at 12,000 rpm for 10 min, the supernatants were discarded, and the pellets were reconstituted in a mixture comprising 180 µL of PBS and 25 µL of Proteinase K (20 g/mL). After vortexing, the samples were incubated for 1 h at 65 °C. HPV DNA extraction was conducted using the commercial CLART HPV2 test kit (Genomica, Madrid, Spain), following the manufacturer’s instructions. The eluted DNAs (100 µL) were stored at −20 °C until further use. 

### 2.4. HPV Genotyping

HPV detection and genotyping were performed using the commercial CLART HPV2 test kit. Biotinylated primers were used to amplify a 450 bp fragment in the HPV L1 region. Co-amplification of an 892 bp fragment of the cystic fibrosis transmembrane conductance regulator (*CFTR*) gene and a 1202 bp fragment of the transformed plasmid served as controls to ensure DNA extraction adequacy and polymerase chain reaction (PCR) efficiency. The amplicons were detected through hybridization in a low density microarray with specific DNA probes to 35 HPV types (6, 11, 16, 18, 26, 31, 33, 35, 39, 40, 42, 43, 44, 45, 51, 52, 53, 54, 56, 58, 59, 61, 62, 66, 68, 70, 71, 72, 73, 81, 82, 83, 84, 85, and 89). The results were obtained using an automatic reader.

In brief, 5 μL of purified DNA was used for PCR amplification, and the PCR products were labeled with biotin during this procedure. The PCR products were then denatured at 95 °C for 10 min and visualized using 5 μL of denatured PCR products. These products hybridized with their respective specific probes immobilized in specific and known regions of the microarray. When the streptavidin conjugate was bound to the biotin-labeled PCR products, an insoluble peroxidase precipitate was formed. The precipitate was analyzed using a Clinical Array Reader (Genomica). Samples with invalid outcomes were retested, and the second result was considered conclusive.

### 2.5. Statistical Analyses 

Descriptive analysis of the epidemiological characteristics of the study participants was conducted. Frequencies and percentages were calculated for the qualitative variables. The age at first intercourse was compared between the vaccinated and unvaccinated groups using Student’s *t*-test. Associations between the proportions were assessed using the chi-square test. A significance level of *p* < 0.05 was applied to all data analyses and relationships between variables. The statistical program used for data processing was Epi Info 7 version 7.2.2.16 (EPI INFO, Centers for Disease and Prevention, Atlanta, GA, USA). Notably, this analysis constitutes part of a broader study focused on detecting changes in HPV prevalence after the implementation of HPV vaccination in Paraguay.

### 2.6. Ethical Considerations

The study was previously examined and authorized by the Institutional Ethics Committee of the Minister of Public Health and Social Welfare’s Central Laboratory of Public Health (Protocol Code CEI-LCSP 152/240419 and Resolutions 110/2019 and 211/2022). The participants signed a free informed consent form before inclusion in the study. Given that primary screening in Paraguay primarily targets women aged 30 and above to identify precursor lesions of cervical cancer, particular attention was given to clearly describe the purpose of this research to the participants. HPV testing was complemented by comprehensive health education efforts designed to inform women and alleviate unnecessary anxiety and distress. The educational initiatives aimed to enhance the understanding of HPV and its connection to cervical cancer, as well as the recommended age for initiating screening.

## 3. Results

### 3.1. Demographic Characteristics of the Study Participants

A total of 254 sexually active young women participated in this study after completing the consent form and questionnaire. The mean patient age was 22 ± 2 years. Approximately 25% of the women resided in Asunción. Most were not economically active (73%) and comprised unemployed individuals, students, and household workers. The detailed sociodemographic characteristics are presented in Table 1.

### 3.2. Vaccination Status 

In total, 40.9% of the participants received at least one dose of the HPV vaccine, corresponding to 104 of 254 individuals. Among the vaccinated women, 46.2% (48/104) completed all three recommended doses, 27.9% (29/104) received two, and 26.0% (27/104) received only one. It is important to note that all participants included in this study were vaccinated with the quadrivalent HPV vaccine (Gardasil-4) as part of the national immunization program.

### 3.3. Risk and Preventive Behaviors and Vaccination Status

The analysis did not uncover any evidence of an association between HPV vaccination and significant changes in the reported indicators of sexual behaviors among sexually active young women. (Table 2).

### 3.4. Prevalence of HPV and Genital Warts and Vaccination Status

**Figure 1:**
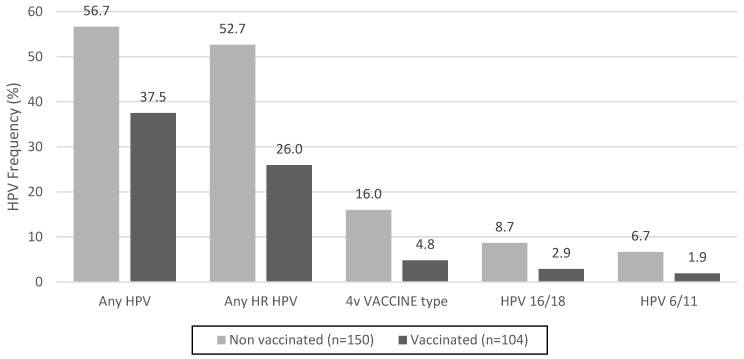
HPV frequency in unvaccinated and vaccinated sexually active young women in Paraguay. High-risk HPV (HR HPV) types include Group 1 IARC, carcinogenic to humans (HPV16, 18, 31, 33, 35, 39, 45, 51, 52, 56, 58, and 59) and Group 2A IARC, probably carcinogenic to humans (HPV68). 4v Vaccine type: HPV quadrivalent vaccine targeting HPV types 6,11,16, and 18.

The prevalence of infection with any HPV type was significantly lower among vaccinated women, at 37.5% [95% confidence interval (CI) 28.2–47.5], compared to 56.7% (95% CI 48.3–64.7) among unvaccinated women (*p* = 0.0026). Similarly, there were significant differences in the prevalence of any high-risk HPV between unvaccinated and vaccinated women [52.7% (95% CI 44.4–60.9) vs. 26.0% (95% CI 17.9–35.5), respectively, *p* < 0.001]. Regarding HPV quadrivalent vaccine types, the frequency decreased in vaccinated women from 16.0% to 4.8% (*p* = 0.01). The data are shown in Figure 1.

A total of twenty-nine different viral types were identified that were present in both single and multiple infections among unvaccinated women. The most commonly detected HPV types were HPV 58 [10.7% (95% CI 6.2–16.7)], followed by HPV 16, HPV 51, and HPV 66 at similar frequencies [7.3% (95% CI 3.7–17.7)]. The distribution of HPV genotypes in positive cases among sexually active unvaccinated women can be seen in Table 3.

In contrast, among the vaccinated women, twenty-three different viral types were identified in single and multiple infections. The most detected HPV type was HPV 51 [6.8% (95% CI 2.8–13.5)], followed by HPV 61 and HPV 70 at similar frequencies [5.8% (95% CI 2.2–12.3)], and HPV 62 [4.9% (95% CI 1.6–11.0)].

Although statistical significance (*p* = 0.0564) was not achieved, there was a noticeable decline in the occurrence of genital warts among vaccinated women when compared to their unvaccinated counterparts. The rates decreased from 20.7% (95% CI 14.5–28.0) to 11.5% (95% CI 6.1–19.3), respectively.

## 4. Discussion

This study is the first exploration of the impact of HPV vaccination by directly comparing the sexual behaviors of vaccinated and unvaccinated young women, focusing on sexually active Paraguayan women aged 18–25 years with the complete information of their HPV vaccination status. No significant difference was observed in the sexual behaviors of young women between those who were HPV-vaccinated and those who were not, adding to the growing body of evidence that HPV vaccination does not encourage changes in sexual behavior among young adults.

Our findings support a consistent pattern across studies, indicating that HPV vaccination does not increase the risk of contracting sexually transmitted infections (STIs), as reported in large retrospective studies based on medical databases that found no correlation between HPV vaccination and increased STI rates [23,30]. Further, clinic-based studies found no difference in STI rates between vaccinated and unvaccinated individuals [31,32,33,34], and population-based studies found no association between risky sexual behaviors or reported rates of STI-related services and HPV vaccination status [35,36,37].

In 2013, a quadrivalent prophylactic HPV vaccine (Gardasil-4) was incorporated into the national immunization program in Paraguay. It was specifically recommended for girls aged 9–14 years and administered in two doses administered at 0 and 6 months. Despite the well-established efficacy and safety of this vaccine [38], Paraguay faces challenges in achieving optimal coverage and initiation rates among adolescents. In 2022, the WHO reported that the first dose of HPV vaccination reached only 45% of the target cohort, and by the age of 15 years, approximately 77% of women had received the vaccine [39]. These statistics underscore the notable gap between the recommended coverage and the actual uptake of the HPV vaccine in Paraguay, emphasizing the importance of addressing barriers to vaccination and enhancing public awareness to achieve more comprehensive protection against HPV-related diseases.

The low uptake of the HPV vaccine can be attributed to a variety of factors, including general barriers to vaccine acceptance among adolescents and specific concerns related to the nature of the vaccine. Common challenges include infrequent visits to healthcare providers, misconceptions regarding vaccine safety, parental attitudes, and missed opportunities for vaccine administration [40,41,42]. Additionally, the fact that HPV is a sexually transmitted infection contributes to parental apprehension with concerns that HPV vaccination might inadvertently encourage children to engage in sexual activity or promote promiscuous and risky sexual conduct. Numerous studies examining parental perspectives on the HPV vaccine have revealed that as many as 20% of parents believe that immunization may lead to riskier sexual behaviors in the future [20]. These multifaceted factors underscore the complexity of overcoming barriers to HPV vaccination, emphasizing the need for targeted interventions addressing both general vaccine hesitancy and specific concerns associated with the HPV vaccine.

As reported in previous studies, no differences were observed in age at sexual debut [20,25,35], and in a Colombian cross-sectional study involving young women aged 18–26 years, we found that the initiation of sexual activity before 15 years was not associated with vaccination status [36]. There was no difference in the mean number of sexual partners between vaccinated and unvaccinated women, consistent with numerous previous studies that examined whether HPV vaccination was associated with a greater number of sexual partners [25,33,35,36,43], which is consistent with a study comparing a sample of vaccinated girls to an unvaccinated historic cohort in the United States that did not find a difference in the mean number of sex partners over the last year or mean number of sex partners in the prior 2 months [31]. Numerous studies have revealed that HPV vaccination is not an important determinant of self-reported sexual activity, and we discovered that vaccination status was not associated with increasing episodes of oral sex [33,34,37,44,45].

Previous studies have shown that the relationship between HPV vaccination status and condom use among young women reveals a complex picture; some research in both community and collegiate settings shows that vaccinated people are more likely to use condoms regularly [31,36], while others show no significant differences in condom usage between vaccinated and unvaccinated groups [24,33,35,43,46,47]. We found no significant differences in condom use between vaccinated and unvaccinated individuals. The diversity in outcomes across various countries highlights that the utilization of condoms is influenced by behavioral factors and not associated with vaccination status.

Interestingly, a notable decline of 19.2% in HPV prevalence rates, closely linked to HPV immunization, was observed, despite the absence of discernible differences in sexual behaviors between vaccinated and non-vaccinated women. This observation aligns with the early indicators of vaccine impact, a trend that is well-documented in numerous studies conducted globally. Several countries in this region have implemented monitoring frameworks to assess the impact of HPV vaccination. Early results indicate a decrease in HPV16/18 prevalence. Notable findings include a study in Argentina, which showed a decline in overall HPV infection and specific infections among vaccinated girls aged 15–17 years [48]. In Brazil, a larger study demonstrated a nearly 57% reduction in HPV types 6, 11, 16, and 18 among vaccinated women aged 16–25 years [49]. These reductions align with clinical trial findings and are consistent with similar outcomes in Australia following HPV vaccine introduction [50]. Accumulating evidence is expected to play a crucial role in demonstrating the effectiveness and impact of HPV vaccination in reducing and eventually eliminating the risk of cervical cancer [51].

Following immunization, countries that use the quadrivalent or nonavalent HPV vaccines should experience a decrease in the occurrence of anogenital warts. In the period studied and considering that the quadrivalent HPV vaccine has been introduced in Paraguay, our results suggest a weak association between HPV vaccination and a decrease in the frequency of genital warts, approaching statistical significance (*p* = 0.0564). However, following vaccinated women is important to observe whether the decline will occur. Since the initiation of quadrivalent HPV vaccination, countries with vaccination programs have experienced substantial reductions in genital warts [52,53,54,55,56,57,58]. Australia has reported a faster and larger-than-expected reduction in genital warts, with a significant 92% reduction [52,53,54]. A Belgian study reported an 8.1% decrease in the general population, with the most significant reduction observed in 16–22 years old participants [59]. These findings underscore the global pattern of significant decreases in genital warts following the implementation of quadrivalent HPV vaccination.

As expected, the infection rate among young women who received the vaccination was noticeably lower, indicating a 20% reduction compared to their unvaccinated counterparts. It is crucial to emphasize that HPV vaccines do not guarantee 100% efficacy against all HPV infections. Current vaccines primarily target the most prevalent high-risk HPV types, specifically HPV 16 and 18, which are strongly associated with cervical cancer development. However, numerous other HPV types still have the potential to cause infections. Within the vaccinated cohort studied, 36% of the participants exhibited HPV infection. Breakthrough infections can occur even in vaccinated women, signifying that they may still contract HPV, although with a reduced likelihood of the infection progressing to a severe disease. While the vaccine does not confer complete immunity, it significantly diminishes the risk of infection with the targeted HPV types [48,49,50,51,60,61].

A literature review extensively examined the impact and effectiveness of HPV vaccination, including substantial reductions in HPV infections, anogenital warts, and cervical lesions across diverse regions, highlighting the global efficacy of vaccination programs. It also emphasized the importance of gender-neutral vaccination, demonstrating its resilience and superior herd protection effects, especially in comparison with female-only programs. Given the widespread circulation of HPV genotypes targeted by the nonavalent vaccine, integrating it into national immunization programs is crucial for achieving broader coverage, especially considering the estimated preventable proportion of cervical cancers reaching 90%. Finally, it underscores the evolving landscape of HPV vaccination, addresses diverse populations, and advocates for strategic planning to maximize its impact on global public health [62].

The difference in prevalent genotypes between unvaccinated and vaccinated women suggests a significant impact of the quadrivalent vaccine against HPV. This supports the consideration of the nonavalent vaccine in national immunization programs. The nonavalent vaccine offers broad protection against genotypes associated with genital warts and cervical cancer, which could drastically reduce the incidence and mortality of this disease, especially in countries with a high burden of cervical cancer. Epidemiological and cost effectiveness studies are needed for countries to make informed decisions on the type of vaccine used in their national immunisation program. It is imperative for individuals who have received the HPV vaccination to adhere to recommended screenings and health checkups, as advised by healthcare professionals. Regular screening combined with vaccination is an essential element of a comprehensive strategy aimed at preventing and managing HPV-related diseases. These findings highlight critical considerations in the strategy of HPV vaccination, including aspects such as partial protection, duration of protection, population-level impact, and the potential for reduced severity of HPV-related diseases [63,64,65].

In a previous study conducted by our research group, representing the primary comprehensive report on HPV prevalence and genotypic distribution in Paraguay, we substantiated the existence of HPV types targeted by contemporary vaccines circulating among unvaccinated, sexually active young women. This established baseline serves as a pivotal tool for evaluating the impact of the national HPV immunization program, facilitating the estimation of both vaccine and non-vaccine HPV type prevalence [66]. Understanding the risk behaviors associated with HPV infection is important for prevention and public health. This study highlights some key points related to risk behaviors and HPV types that are crucial for the progression and elimination of cervical cancer.

Despite the valuable insights provided, our study has several notable limitations. One of the major constraints is the reliance on self-reported vaccination history, introducing the possibility of both overreporting and underreporting. Additionally, the use of self-administered questionnaires to collect information on risk factors raises concerns about data accuracy. Regarding genital warts, a significant aspect of the quadrivalent vaccine’s contribution, encompassing low-risk HPV genotypes primarily linked to this HPV-associated condition, it is crucial to emphasize that the analysis of genital warts history in this study may be biased due to reliance solely on self-reports from participants. To address these limitations and ensure a more robust evaluation, future studies should consider incorporating national data on the prevalence, incidence, and treatment rates of genital warts. Another substantial constraint is the relatively small sample size, limiting the generalizability of specific analyses. Additionally, it is important to note that the patients mainly originated from the capital and its nearby regions. Nonetheless, larger-scale research endeavors in the future, involving a more diverse participant pool, will provide valuable opportunities to systematically assess the impact of vaccination on the prevalence of various HPV types. This includes examining vaccine types, potential cross-protection, and the potential for genotype replacement. Moreover, efforts to validate self-reported vaccination history through medical records or other reliable sources could enhance the accuracy of the findings. Implementing these improvements will contribute to a more comprehensive understanding of the effectiveness and implications of HPV vaccination.

## 5. Conclusions

The vaccination against the HPV has had a considerable and demonstrable impact on the prevention of HPV-related diseases worldwide. In 2013, Paraguay incorporated the quadrivalent prophylactic HPV vaccine, Gardasil-4, into its national immunization program. The low uptake of the HPV vaccine is influenced by various factors, including infrequent healthcare visits, misconceptions about vaccine safety, and parental concerns that administering the HPV vaccine might promote early onset of sexual activity or promiscuous and risky behavior. This study adds to the growing body of evidence indicating that HPV vaccination does not influence the sexual behavior of Paraguayan young women. By underscoring the significance of physician recommendations and parental approval in fostering vaccine acceptance, our study highlights the importance of supporting evidence that demonstrates no association between HPV vaccination and subsequent changes in sexual behavior. Given the long latency period between HPV infection and progression to cancer, the full impact of HPV vaccination in Paraguay cannot be observed completely yet. However, these data affirm the positive early impact of HPV vaccination among Paraguayan young women, making a significant contribution to the reduction in overall HPV infection rates and its associated lesions. Additionally, recognizing the significance of testing for all types of HPV infections in vaccinated women is essential. Considering these findings, it is crucial to emphasize the importance of cervical screening and understanding HPV-associated diseases as integral components of comprehensive risk management strategies.

## Figures and Tables

**Table 1:** Sociodemographic characteristics of study population (*n* = 254).

Characteristics	Study Population (*n* = 254)			
	N	% or Mean	95% Confidence Interval	
Age (mean years)		22.1 ± 2.2	21.8	22.4
Residency				
Asunción	63	24.8	19.6	30.6
Other localities	191	75.2	69.4	80.4
Education level				
Primary/Secondary	70	27.6	22.2	33.5
Tertiary	184	72.4	66.5	77.8
Occupation				
Economically active (have any job)	69	27.2	21.8	33.1
Not economically active (unemployed, student, houseworkers)	185	72.8	66.9	78.2
Live with				
Parents/Friends/Alone	211	83.1	77.9	87.5
Partner	43	16.9	12.5	22.1

**Table 2:** Differences in risk and preventive behaviors among HPV-vaccinated and unvaccinated sexually active young women (*n* = 254).

Variable	Vaccination Status		*p* Value
	Yes *n* (%)/Mean (SD) (*n* = 104)	No *n* (%)/Mean (SD) (*n* = 150)	
*Sexual behavior*			
Age at sexual debut (mean years)	16.8 ± 2.2	16.7 ± 2.1	0.9744
≤15 years	27 (26.0)	38 (25.3)	0.9102
>15 years	77 (74.0)	112 (74.7)	
Sex partners in the past 12 months			
≥2	39 (37.5)	65 (43.3)	0.3525
≤1	65 (62.5)	85 (56.7)	
Oral sex			
Yes	92 (86.5)	131 (87.3)	0.7871
No	12 (11.5)	19 (12.7)	
Hormonal contraceptives			
Yes	30 (28.9)	41 (27.3)	0.7916
No	74 (71.2)	109 (72.7)	
Condom use			
Less than every time	76 (73.1)	112 (74.7)	0.7763
Every time	28 (26.9)	38 (25.3)	
History of any sexually transmitted disease			
Yes	24 (23.1)	50 (33.3)	0.0768
No	80 (76.9)	100 66.7)	
History of anogenital warts			
Yes	12 (11.5)	31 (20.7)	0.0564
No	92 (88.5)	119 (79.3)	
HPV infection			
Yes	39 (37.5)	85 (56.7)	0.0026
No	65 (62.5)	65 (43.3)	
*Smoking status*			
Yes	18 (17.3)	33 (22.0)	0.3586
No	86 (82.7)	117 (78.0)	
*Alcohol use*			
Yes	77 (74.0)	123 (82.0)	0.1273
No	27 (26.0)	27 (18.0)	

SD, Standard deviation.

**Table 3:** Distribution of viral genotype among HPV-positive non-vaccinated women from Paraguay. (*n* = 85).

	Genotype	Cases (*n*)	Frequency (%) *n* = 85
LR-HPV	6	8	9.4
	11	2	2.4
	40	2	2.4
	42	5	5.9
	54	2	2.4
	61	2	2.4
	71	2	2.4
	72	1	1.2
	81	8	9.4
	83	2	2.4
	84	2	2.4
pHR-HPV	26	2	2.4
	53	11	12.9
	66	9	10.6
	70	5	5.9
	82	4	4.7
	85	1	1.2
HR-HPV	16	11	12.9
	18	3	3.5
	31	8	9.4
	33	8	9.4
	35	5	5.9
	39	1	1.2
	45	1	1.2
	51	11	12.9
	52	9	10.6
	56	1	1.2
	58	16	18.8
	59	5	5.9

HR-HPV: High risk HPV types; pHR-HPV: possible high risk; and LR-HPV: low risk HPV types. HR types, including Group 1 IARC, carcinogenic to humans (HPV16, 18, 31, 33, 35, 39,45, 51, 52, 56, 58, and 59) and Group 2A IARC, probably carcinogenic to humans (HPV68). pHR types, Group 2B IARC, possibly carcinogenic to humans (HPV26, 30, 34, 53, 66, 67, 69, 70, 73, 82, 85, and 97). LR types, non-carcinogenic to humans (HPV6, 11, 40, 42, 54, 61, 71, 72, 81, 83, and 84).

## Data Availability

The data presented in this study are available upon request from the corresponding author.

## References

[1] Ferlay J., Ervik M., Lam F., Colombet M., Mery L., Piñeros M., Znaor A., Soerjomataram I., Bray F. (2020). Global Cancer Observatory: Cancer Today. Lyon, France: International Agency for Research on Cancer. https://gco.iarc.fr/today.

[2] World Health Organization (2022). Human Papillomavirus Vaccines. WHO Position Paper.

[3] Villa L.L., Costa R.L., Petta C.A., Andrade R.P., Ault K.A., Giuliano A.R., Wheeler C.M., Koutsky L.A., Malm C., Lehtinen M. (2005). Prophylactic quadrivalent human papillomavirus (types 6, 11, 16, and 18) L1 virus-like particle vaccine in young women: A randomised double-blind placebo-controlled multicentre phase II efficacy trial. Lancet Oncol..

[4] Garland S.M., Hernandez-Avila M., Wheeler C.M., Perez G., Harper D.M., Leodolter S., Tang G.W., Ferris D.G., Steben M., Bryan J. (2007). Quadrivalent vaccine against human papillomavirus to prevent anogenital diseases. N. Engl. J. Med..

[5] Harper D.M., Franco E.L., Wheeler C.M., Moscicki A.B., Romanowski B., Roteli-Martins C.M., Jenkins D., Schuind A., Costa Clemens S.A., Dubin G. (2006). Sustained efficacy up to 4.5 years of a bivalent L1 virus-like particle vaccine against human papillomavirus types 16 and 18: Follow-up from a randomised control trial. Lancet.

[6] Harper D.M., Franco E.L., Wheeler C., Ferris D.G., Jenkins D., Schuind A., Zahaf T., Innis B., Naud P., De Carvalho N.S. (2004). Efficacy of a bivalent L1 virus-like particle vaccine in prevention of infection with human papillomavirus types 16 and 18 in young women: A randomised controlled trial. Lancet.

[7] Paavonen J., Jenkins D., Bosch F.X., Naud P., Salmeron J., Wheeler C.M., Chow S.N., Apter D.L., Kitchener H.C., Castellsague X. (2007). Efficacy of a prophylactic adjuvanted bivalent L1 virus-like-particle vaccine against infection with human papillomavirus types 16 and 18 in young women: An interim analysis of a phase III double-blind, randomised controlled trial. Lancet.

[8] Lukács A., Máté Z., Farkas N., Mikó A., Tenk J., Hegyi P., Németh B., Czumbel L.M., Wuttapon S., Kiss I. (2020). The quadrivalent HPV vaccine is protective against genital warts: A meta-analysis. BMC Public Health.

[9] Joura E.A., Leodolter S., Hernandez-Avila M., Wheeler C.M., Perez G., Koutsky L.A., Garland S.M., Harper D.M., Tang G.W., Ferris D.G. (2007). Efficacy of a quadrivalent prophylactic human papillomavirus (types 6, 11, 16, and 18) L1 virus-like-particle vaccine against high-grade vulval and vaginal lesions: A combined analysis of three randomized clinical trials. Lancet.

[10] Schiller J.T., Castellsague X., Villa L.L., Hildesheim A. (2008). An update of prophylactic human papillomavirus L1 virus-like particle vaccine clinical trial results. Vaccine.

[11] Luckett R., Feldman S. (2016). Impact of 2-, 4- and 9-valent HPV vaccines on morbidity and mortality from cervical cancer. Hum. Vaccines Immunother..

[12] Shu Y., Yu Y., Ji Y., Zhang L., Li Y., Qin H., Huang Z., Ou Z., Huang M., Shen Q. (2022). Immunogenicity and safety of two novel human papillomavirus 4- and 9-valent vaccines in Chinese women aged 20-45 years: A randomized, blinded, controlled with Gardasil (type 6/11/16/18), phase III non-inferiority clinical trial. Vaccine.

[13] World Health Organization (WHO) Immunization, Vaccines and Biologicals. HPV Dashboard. https://www.who.int/teams/immunization-vaccines-and-biologicals/diseases/human-papillomavirus-vaccines-(HPV)/hpv-clearing-house/hpv-dashboard.

[14] World Health Organization (2017). Human Papillomavirus Vaccines: WHO Position Paper. Weekly Epidemiological Record. WER No 19.

[15] (2017). Normas Nacionales de Vacunación, Técnico Administrativas y de Vigilancia del Programa Nacional de Enfermedades Inmunoprevenibles y PAI.

[16] Brisson M., Bénard É., Drolet M., Bogaards J.A., Baussano I., Vänskä S., Jit M., Boily M.-C., Smith M., Berkhof J. (2016). Population-level impact, herd immunity, and elimination after human papillomavirus vaccination: A systematic review and meta-analysis of predictions from transmission-dynamic models. Lancet Public Health.

[17] Bruni L., Diaz M., Barrionuevo-Rosas L., Herrero R., Bray F., Bosch F.X., de Sanjosé S., Castellsagué X. (2016). Global estimates of human papillomavirus vaccination coverage by region and income level: A pooled analysis. Lancet Glob. Health.

[18] Bird Y., Obidiya O., Mahmood R., Nwankwo C., Moraros J. (2017). Human papillomavirus vaccination uptake in Canada: A systematic review and meta-analysis. Int. J. Prev. Med..

[19] Smith A., Baines N., Memon S., Fitzgerald N., Chadder J., Politis C., Nicholson E., Earle C., Bryant H. (2019). Moving toward the elimination of cervical cancer: Modelling the health and economic benefits of increasing uptake of human papillomavirus vaccines. Curr. Oncol..

[20] Forster A., Wardle J., Stephenson J., Waller J. (2020). Passport to promiscuity or lifesaver: Press coverage of HPV vaccination and risky sexual behavior. J. Health Commun..

[21] Schuler C.L., Reiter P.L., Smith J.S., Brewer N.T. (2011). Human papillomavirus vaccine and behavioral disinhibition. Sex. Transm. Infect..

[22] Holman D.M., Benard V., Roland K.B., Watson M., Liddon N., Stokley S. (2014). Barriers to human papillomavirus vaccination among US adolescents: A systematic review of the literature. JAMA Pediatr..

[23] Bednarczyk R.A., Davis R., Ault K., Orenstein W., Omer S.B.J. (2012). Sexual activity–related outcomes after human papillomavirus vaccination of 11-to 12-year-olds. Pediatrics.

[24] Forster A.S., Marlow L.A., Stephenson J., Wardle J., Waller J. (2012). Human papillomavirus vaccination and sexual behavior: Cross-sectional and longitudinal surveys conducted in England. Vaccine.

[25] Hansen B.T., Kjær S.K., Arnheim-Dahlström L., Liaw K.-L., Jensen K.E., Thomsen L.T., Munk C., Nygård M. (2014). Human papillomavirus (HPV) vaccination and subsequent sexual behavior: Evidence from a large survey of Nordic women. Vaccine.

[26] Smith L.M., Kaufman J.S., Strumpf E.C., Levesque L.E. (2015). Effect of human papillomavirus (HPV) vaccination on clinical indicators of sexual behavior among adolescent girls: The Ontario Grade 8 HPV Vaccine Cohort Study. Can. Med. Assoc. J..

[27] Madhivanan P., Pierre-Victor D., Mukherjee S., Bhoite P., Powell B., Jean-Baptiste N., Clarke R., Avent T., Krupp K. (2016). Human papillomavirus vaccination and sexual disinhibition in females: A systematic review. Am. J. Prev. Med..

[28] Mullins T.L.K., Zimet G.D., Rosenthal S.L., Morrow C., Ding L., Huang B., Kahn J.A. (2016). Human papillomavirus vaccine-related risk perceptions and subsequent sexual behaviors and sexually transmitted infections among vaccinated adolescent women. Vaccine.

[29] Ogilvie G.S., Phan F., Pedersen H.N., Dobson S.R., Naus M., Saewyc E.M. (2018). Population-level sexual behaviors in adolescent girls before and after introduction of the human papillomavirus vaccine (2003–2013). Can. Med. Assoc. J..

[30] Jena A.B., Goldman D.P., Seabury S.A. (2015). Incidence of sexually transmitted infections after human papillomavirus vaccination among adolescent females. JAMA Intern. Med..

[31] Cummings T., Zimet G.D., Brown D., Tu W., Yang Z., Fortenberry J.D., Shew M.L. (2012). Reduction of HPV infections through vaccination among at-risk urban adolescents. Vaccine.

[32] Lutringer-Magnin D., Kalecinski J., Cropet C., Barone G., Ronin V., Régnier V., Leocmach Y., Jacquard A.-C., Vanhems P., Chauvin F. (2013). Prevention of sexually transmitted infections among girls and young women in relation to their HPV vaccination status. Eur. J. Public Health.

[33] Rysavy M.B., Kresowik J.D., Liu D., Mains L., Lessard M., Ryan G.L. (2014). Human papillomavirus vaccination and sexual behavior in young women. J. Pediatr. Adolesc. Gynecol..

[34] Sadler L., Roberts S.A., Hampal G., McManus D., Mandal D., Brabin L. (2015). Comparing risk behaviours of human papillomavirus-vaccinated and non-vaccinated women. J. Fam. Plann. Reprod. Health Care.

[35] Liddon N.C., Leichliter J.S., Markowitz L.E. (2012). Human papillomavirus vaccine and sexual behavior among adolescent and young women. Am. J. Prev. Med..

[36] Ruiz-Sternberg A.M., Pinzon-Rondon A.M. (2014). Risk perception and sexual behavior in HPV-vaccinated and unvaccinated young Colombian women. Int. J. Gynaecol. Obstet..

[37] Mattebo M., Grün N., Rosenblad A., Larsson M., Häggström-Nordin E., Dalianis T., Tydén T. (2014). Sexual experiences in relation to HPV vaccination status in female high school students in Sweden. Eur. J. Contracept. Reprod. Health Care.

[38] Kamolratanakul S., Pitisuttithum P. (2021). Human Papillomavirus Vaccine Efficacy and Effectiveness against Cancer. Vaccines.

[39] World Health Organization (WHO) (2023). Human Papillomavirus (HPV) Vaccination Coverage. OMS: Switzerland. https://immunizationdata.who.int/pages/coverage/hpv.html?CODE=amr.

[40] Roberts J.R., Thompson D., Rogacki B., Hale J.J., Jacobson R.M., Opel D.J., Darden P.M. (2015). Vaccine hesitancy among parents of adolescents and its association with vaccine uptake. Vaccine.

[41] Kornides M.L., Garrell J.M., Gilkey M.B. (2017). Content of web-based continuing medical education about HPV vaccination. Vaccine.

[42] Brouwer A.F., Delinger R.L., Eisenberg M.C., Campredon L.P., Walline H.M., Carey T.E., Meza R. (2019). HPV vaccination has not increased sexual activity or accelerated sexual debut in a college-aged cohort of men and women. BMC Public Health.

[43] Marchand E., Glenn B.A., Bastani R. (2013). HPV vaccination and sexual behavior in a community college sample. J. Community Health.

[44] Mather T., McCaffery K., Juraskova I. (2012). Does HPV vaccination affect women’s attitudes to cervical cancer screening and safe sexual behavior?. Vaccine.

[45] Aujo J.C., Bakeera-Kitaka S., Kiguli S., Mirembe F. (2014). No difference in sexual behavior of adolescent girls following human papillomavirus vaccination: A case study two districts in Uganda; Nakasongola and Luwero. BMC Public Health.

[46] Sopracordevole F., Cigolot F., Mancioli F., Agarossi A., Boselli F., Ciavattini A. (2013). Knowledge of HPV infection and vaccination among vaccinated and unvaccinated teenaged girls. Int. J. Gynaecol. Obstet..

[47] Ratanasiripong N.T. (2014). Human papillomavirus vaccine increases high-risk sexual behaviors: A myth or valid concern. J. Sch. Nurs..

[48] González J.V., DeLuca G.D., Liotta D.J., Correa R.M., Basiletti J.A., Colucci M.C., Katz N., Vizzotti C., Picconi M.A., MALBRAN HPV Surveillance Study Group (2021). Baseline prevalence and type distribution of Human papillomavirus in sexually active non-vaccinated adolescent girls from Argentina. Rev. Argent. Microbiol..

[49] Wendland E.M., Kops N.L., Bessel M., Comerlato J., Maranhão A.G.K., Souza F.M.A., Villa L.L., Pereira G.F.M. (2021). Effectiveness of a universal vaccination program with an HPV quadrivalent vaccine in young Brazilian women. Vaccine.

[50] Tabrizi S.N., Brotherton J.M.L., Kaldor J.M., Skinner S.R., Liu B., Bateson D., McNamee K., Garefalakis M., Phillips S., Cummins E. (2014). Assessment of herd immunity and cross-protection after a human papillomavirus vaccination programme in Australia: A repeat cross-sectional study. Lancet Infect. Dis..

[51] García-Perdomo H.A., Osorio J.C., Fernandez A., Zapata-Copete J.A., Castillo A. (2019). The effectiveness of vaccination to prevent the papillomavirus infection: A systematic review and meta-analysis. Epidemiol. Infect..

[52] Donovan B., Franklin N., Guy R., Grulich A.E., Regan D.G., Ali H., Wand H., Fairley C.K. (2011). Quadrivalent human papillomavirus vaccination and trends in genital warts in Australia: Analysis of national sentinel surveillance data. Lancet Infect. Dis..

[53] Read T.R.H., Hocking J.S., Chen M.Y., Donovan B., Bradshaw C.S., Fairley C.K. (2011). The near disappearance of genital warts in young women 4 years after commencing a national human papillomavirus (HPV) vaccination programme. Sex. Transm. Infect..

[54] Ali H., Donovan B., Wand H., Read T.R.H., Regan D.G., Grulich A., Fairley C.K., Guy R.J. (2013). Genital warts in young Australians five years into national human papillomavirus vaccination programme: National surveillance data. BMJ.

[55] Baandrup L., Blomberg M., Dehlendorff C., Sand C., Andersen K.K., Kjaer S.K. (2013). Significant decrease in the incidence of genital warts in young Danish women after implementation of a national human papillomavirus vaccination program. Sex. Transm. Dis..

[56] Blomberg M., Dehlendorff C., Munk C., Kjaer S.K. (2013). Strongly decreased risk of genital warts after vaccination against human papillomavirus: Nationwide follow-up of vaccinated and unvaccinated girls in Denmark. Clin. Infect. Dis..

[57] Dochez C., Bogers J.J., Verhelst R., Rees H. (2014). HPV vaccines to prevent cervical cancer and genital warts: An update. Vaccine.

[58] Bollerup S., Baldur-Felskov B., Blomberg M., Baandrup L., Dehlendorff C., Kjaer S.K. (2016). Significant Reduction in the Incidence of Genital Warts in Young Men 5 Years into the Danish Human Papillomavirus Vaccination Program for Girls and Women. Sex. Transm. Dis..

[59] Dominiak-Felden G., Gobbo C., Simondon F. (2015). Evaluating the Early Benefit of Quadrivalent HPV Vaccine on Genital Warts in Belgium: A Cohort Study. PLoS ONE.

[60] Puerto D., Reyes V., Lozano C., Buitrago L., Garcia D., Murillo R.H., Muñoz N., Hernandez G.A., Sanchez L., Wiesner C. (2018). Detection and Genotyping of HPV DNA in a Group of Unvaccinated Young Women from Colombia: Baseline Measures Prior to Future Monitoring Program. Cancer Prev. Res..

[61] Dunne E.F., Klein N.P., Naleway A.L., Baxter R., Weinmann S., Riedlinger K., Fetterman B., Steinau M., Scarbrough M.Z., Gee J. (2013). Prevalence of HPV types in cervical specimens from an integrated healthcare delivery system: Baseline assessment to measure HPV vaccine impact. Cancer Causes Control.

[62] Wang W.V., Kothari S., Skufca J., Giuliano A.R., Sundström K., Nygård M., Koro C., Baay M., Verstraeten T., Luxembourg A. (2022). Real-world impact and effectiveness of the quadrivalent HPV vaccine: An updated systematic literature review. Expert. Rev. Vaccines.

[63] Herweijer E., Feldman A.L., Ploner A., Arnheim-Dahlström L., Uhnoo I., Netterlid E., Dillner J., Sparén P., Sundström K. (2015). The Participation of HPV-Vaccinated Women in a National Cervical Screening Program: Population-Based Cohort Study. PLoS ONE.

[64] El-Zein M., Richardson L., Franco E.L. (2016). Cervical cancer screening of HPV vaccinated populations: Cytology, molecular testing, both or none. J. Clin. Virol..

[65] Thomas C., Wright T.C., Valentin Parvu V., Mark H., Stoler M.H., Salma Kodsi S., Karen Eckert K., Karen Yanson K., Charles K., Cooper C.K. (2019). HPV infections and cytologic abnormalities in vaccinated women 21–34 years of age: Results from the baseline phase of the Onclarity trial. Gynecol. Oncol..

[66] Bobadilla M.L., Villagra V., Ortiz V., Deluca G., de Paula V.S. (2023). High prevalence and co-infection of high-risk Human Papillomavirus genotypes among unvaccinated young women from Paraguay. PLoS ONE.

